# Clinicopathological Characteristics of *PIK3CA* Mutation and Amplification in Korean Patients with Breast Cancers

**DOI:** 10.7150/ijms.44319

**Published:** 2020-05-01

**Authors:** Moo-Hyun Lee, Ji-Hyung Cho, Sun-Young Kwon, Soo-Jung Jung, Jae-Ho Lee

**Affiliations:** 1 Department of General Surgery, Keimyung University Dongsan Medical Center; 2 Department of Pathology, Keimyung University Dongsan Medical Center; 3 Department of Anatomy, Keimyung University School of Medicine

**Keywords:** breast carcinoma, PIK3CA mutation, PIK3CA amplification

## Abstract

The frequency of *PIK3CA* mutation and amplification was various and their clinical significances have not been clarified in Korean patients with invasive breast carcinoma (IBC). The study aimed to investigate the clinical and prognostic significances of *PIK3CA* mutation and amplification in IBC patients. DNA was isolated from paired normal and tumoral tissues in 128 IBC patients and the mutation and expression of *PIK3CA* gene were analyzed. *PIK3CA* mutation and expression was detected in 14.3% and 21.9% of IBC patients, respectively. And the level of *PIK3CA* expression was not different according to the presence of *PIK3CA* mutation (p = 0.775). *PIK3CA* mutation and expression were significantly associated with Luminal A type (p = 0.017 and p = 0.011, respectively). However, they did not have any clinical and prognostic values for IBC patients. This result suggested that alterations of PIK3CA pathway contribute to the pathogenesis of specific type of IBC.

## Introduction

Invasive breast carcinoma (IBC) is the most frequent cancer in women worldwide and has been studied for many decades; however, its etiology remains unknown 1,2. Many studies have shown that IBC is a complex and multifactorial disease, and its development has been associated with numerous risk factors including age, parity, hormonal influences, lifestyle, environment, diet, race and inherited genetic predisposition 1-3. In the era of next-generation sequencing, several genetic alterations have been focused on pathogenesis of IBC 4-6. Interestingly, these alterations differed according to IBC subtypes and many clinical trials have been proposed based on these results 7,8.

In many ongoing trials, PIK3CA is considered a targetable potential driver of BC. Phosphatidylinositol-3-kinase (*PI3K/Akt*) pathway regulates multiple biological signaling such as cell proliferation, differentiation, apoptosis, and mortality 9. The *PI3Ks* are heterodimers consisting of a catalytic subunit (*p110α, PIK3CA*) and an adaptor/ regulatory subunit (*p85*), subunits that are activated by tyrosine kinase growth factor receptors such as epidermal growth factor receptor (*EGFR*) and insulin-like growth factor-1 receptor (*IGF-1R*), cell adhesion molecules such as integrins, G-protein- coupled receptors (*GPCRs*), and oncogenes such as *Ras* 10. The frequency of *PIK3CA* mutation of IBC in Korean population was high (26.9%), and its mutation and amplification were frequent and important in IBC 11. Therefore, PIK3CA inhibitors are being used to treat IBC according to ER status in clinical trials 12,13. However, both mutation and amplification of PIK3CA gene have not been analyzed in Korean patients with IBC.

In present study, clinical and prognostic values of *PIK3CA* mutation and amplification were investigated in 128 Korean patients with IBC. This study will allow a better understanding and insight into molecular mechanisms of IBC, which have essential implications for IBC therapy.

## Materials and Methods

### Patients and DNA Extraction

One hundred and twenty-eight patients diagnosed with IBC were included in the study. IBCs and adjacent non-neoplastic tissues were obtained from patients undergoing surgery in Keimyung University Dongsan Medical Center (Daegu, Korea) between 2011 and 2014. Tissue samples were provided from the Keimyung Human Bio-Resource Bank, Korea. All patients were educated of the study purpose and informed consent was obtained from each study participant. The protocols were approved by the Institutional Review Board of Keimyung University Dongsan Medical Center (approval #2014-03-038-002).

Data on histopathological characteristics including staging, histologic grade, estrogen receptor (ER), progesterone receptor (PR), and human epidermal growth factor receptor 2 (HER2) were obtained from pathology records. Molecular subtypes were defined based on the St Gallen's criteria on ER, PR, and HER2 in conjunction with histologic grade. The staging of primary tumor was based on the The American Joint Committee on Cancer Staging Manual, 7th edition 14. All markers were visually assessed by pathologists (Kwon SY). Tumor areas and adjacent normal mucosa were used for DNA extraction using an extraction kit (Absolute DNA Extraction Kit, BioSewoom, Gyeongsangnam-do, South Korea) according to the manufacturer's instructions. DNA quantity and quality were measured using a NanoDrop 1000 (Thermo Fisher Scientific, Pittsburgh, PA, USA).

### *PIK3CA* Mutation Analysis

Two hot spot-regions (exons 9 and 20) of *PIK3CA* mutation were investigated in the 50 HCC samples, because more than 75% of *PIK3CA* missense mutations cluster were found within these regions 4-6. The polymerase chain reaction (PCR) amplification of the *PIK3CA* was performed as described previously 9-11. The primer sequences of the *PIK3CA* exon 9 were forward, 5'-CCA GAG GGG AAA AAT ATG ACA A-3' and reverse, 5'-ACC TGT GAC TCC ATA GAA A-3'. The sequences of the exon 20 primers were forward, 5'-TTG ATG ACA TTG CAT ACA TTC G-3' and reverse, 5'-AAT TGT GTG GAA GAT CCA ATC C-3'. PCR was done using AmpliTaq Gold (Applied Biosystems, USA). The PCR conditions were as follows: 1 cycle of 95 ℃ for 11 min, 40 cycles of 95 ℃ for 30 sec, 55 ℃ for 40 sec, and 72 ℃ for 1 min, followed by 1 cycle of 72 ℃ for 10 min. The PCR products were electrophoresed on 1.5% agarose gel and stained with ethidium bromide to confirm the size of the bands. Direct DNA sequencing of *PIK3CA* was subsequently performed using an ABI 3730 DNA sequencer (Bionics, Seoul, South Korea).

### PIK3CA Amplification

Copy number of PIK3CA gene was analyzed by quantitative real-time (qRT) PCR. For the quantitative determination of PIK3CA content relative to nDNA, primers for specific amplification of exon 20 in PIK3CA gene and nDNA-encoded ß-actin gene were selected according to previous study. Real-time PCR was then carried out on an LightCycler 480 II system (Roche Diagnostics, Germany) with a total volume of 20 µl reaction mixture containing 10 µl SYBR Green Master MIX (Takara, Japan), 8 pmol of each primers, and DNA (50 ng). The PCR conditions were 95°C for 1 min, followed by 40 cycles of 95°C for 15 s, and 60°C for 30 s. The threshold cycle number (Ct) values of the ß -actin gene and PIK3CA gene were determined. The copy number of PIK3CA in each tested specimen was then normalized against that of ß -actin gene to calculate the relative PIK3CA copy number. Each measurement was repeated in triplicate and 5 serially diluted control samples were included in each experiment.

### Statistical Analysis

With the SPSS 25.0 (SPSS, Inc., USA), statistical analysis was performed by using the Chi-square test or the Fisher's exact test for categorical variables and the Mann-Whitney U-test for continuous variables. Survival curves, constructed using the univariate Kaplan-Meier estimators, were compared using the log-rank test. Overall survival (OS) was defined as the time between diagnosis and mortality. Disease-free survival (DFS) was defined as the time between diagnosis, and disease recurrence or the development of distant metastasis. *P* values of below 0.05 were considered significant.

## Results

**Figure 1:**
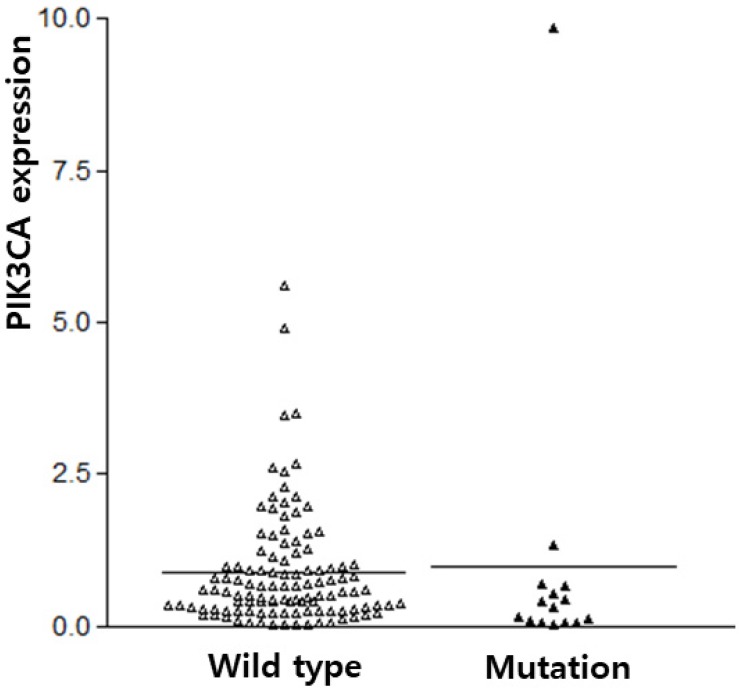
PIK3CA expression according to PIK3CA mutation in breast cancers.

The subjects of this study were 128 IBC patients (54.77 ± 11.85 years old). The sequencing for *PIK3CA* exons 9 and 20 were successfully performed from 105 IBC. *PIK3CA* mutation was found in 14.3% (15/105) of IBC. *PIK3CA* mutations were predominantly found in exon 20 (H1047R, n=11, 73.3%) as compared to exon 9 (E542K and E545K, n=4, 26.7%, with similar frequencies). The *PIK3CA* expression was analyzed in all 128 IBCs. The average *PIK3CA* expression in the IBC tissue was 0.98 ± 0.15, which contrasted with its expression in normal tissues. *PIK3CA* expression was not different according to *PIK3CA* mutation (p = 0.775, Figure 1).

To further explore the correlation between *PIK3CA* expression and the clinicopathological parameters of IBC, patients were categorized into two subgroups according to the average values of the Tumor/Non-tumor ratio (≥ 0.98). *PIK3CA* expression was found in 21.9% (28/128) of IBCs. Clinicopathological characteristics associated with the *PIK3CA* mutation and expression are summarized in Table 1. *PIK3CA* mutation tended to be associated with differentiation, though it did not have a statistical significance (p = 0.089). *PIK3CA* mutation and *PIK3CA* expression was more frequent in Luminal A type and they are absent in HER2+ type (p = 0.017 and p = 0.011, respectively). Other clinicopathological characteristics were not associated with *PIK3CA* mutation and expression.

**Figure 2:**
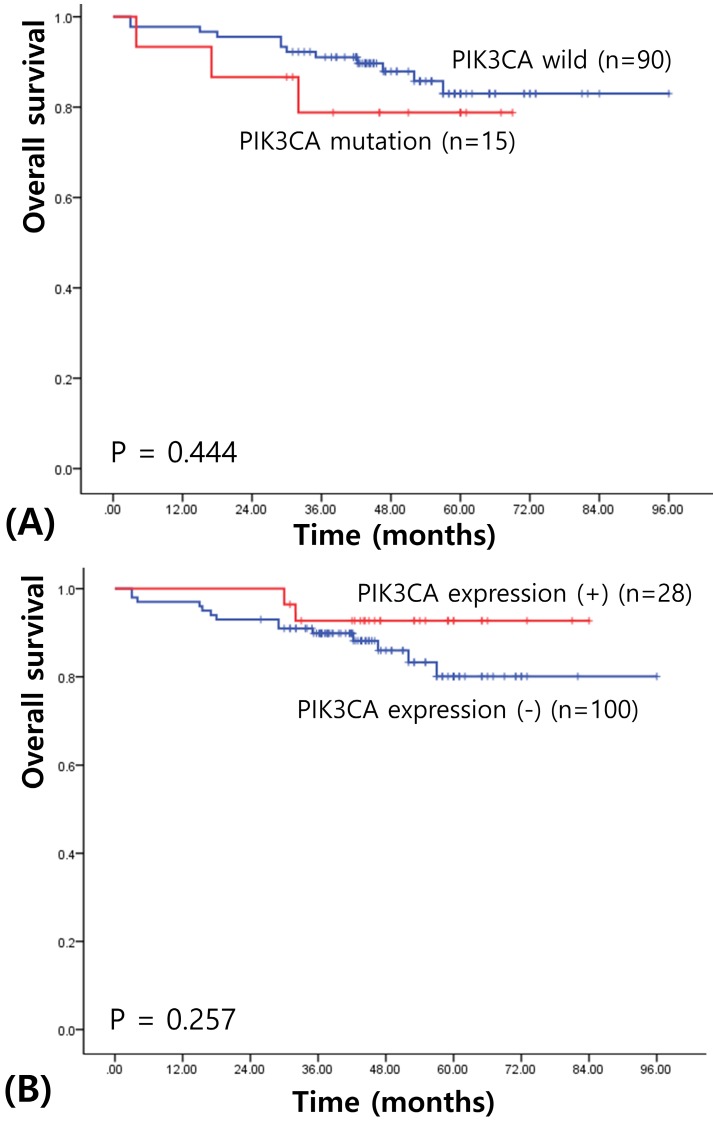
Kaplan-Meier cures for overall survival in breast cancers according to PIK3CA mutation (A) and PIK3CA expression (B).

We next assessed survival to determine the prognostic significances of *PIK3CA* mutation and expression in IBC. The median follow-up of patients for survival analysis was 58.93 months (range, 3-106 months). Univariate survival analysis using Kaplan-Meier curves showed no prognostic value of *PIK3CA* mutation (χ^2^= 0.586, *P* = 0.444; see Figure 2A). PIK3CA expression also did not have any prognostic value for the patients with IBCs (χ^2^= 1.283, *P* = 0.257; see Figure 2B). When stratifying for different variables, *PIK3CA* status did not appear to confer any statistically significant prognostic value.

## Discussion

*PIK3CA* is a key down-stream protein kinase of the PI3K-AKT signaling pathway playing an important role in cancer cell proliferation, catabolism, cell adhesion and apoptosis 15. Previous studies have shown that *PIK3CA*, as a subunit of PI3K, is frequently mutated in various human cancers as a main mechanism in the activation of PI3K/Akt signaling pathway 4-6,9-11. Though most studies have focused the significance of *PIK3CA* mutation, some studies about *PIK3CA* expression in IBC were also performed 16,17. Several characteristics of PIK3CA mutations and expression in IBC have been observed, including a strong association with the estrogen receptor, a lack of an association with robust activation of the classical PI3K pathway, as well as a relatively better prognosis for patients with mutation type compared with wild-type counterparts 9-17.

For a first time, both PIK3CA mutations and expression were studied in Korean patients with IBC. The frequencies of *PIK3CA* mutations (14.3%) were in concordance with previous studies and slightly lower because only two hot spot mutations were analyzed 11,13,14. According to The Cancer Genome Atlas (TCGA) data, *PIK3CA* mutation was found in 34.18% of IBC 6. Detail functions of two mutations, E542K or E545K in exon 9 and H1047R or H1047L in exon 20 were demonstrated 18,19. Exon 9 mutations are located in the helical domain of p110α, and are considered to enable p110α to escape the inhibitory effect of p85 via the Src-homology 2 (SH2) domain. Exon 20 mutations are located near the activation loop in the kinase domain, promoting constitutive PI3K signaling. These mutations may induce a breast carcinogenesis via PI3K activation.

PIK3CA expression level was also in agreement with previous studies 16,17. Like PIK3CA mutations, PIK3CA amplification has been shown to lead to increased PI3K activity 17. We did not find any association between PIK3CA mutation and expression. Several reports on various cancers indicated mutual exclusivity between PIK3CA mutations and PIK3CA amplification, suggesting that each of these alterations may individually be sufficient to drive their carcinogenesis independently 20,21. Interestingly, PIK3CA mutation and expression did not have any clinical significance, except luminal classification. The correlation of PIK3CA and hormone receptor status has been confirmed by many authors 5,6,9. These data and our results suggested that genetic change of PIK3CA tends to be found luminal type tumors, less aggressive characteristics. Clinicopathological characteristics of PIK3CA mutation and expression should be confirmed further with larger cases of IBC.

**Figure 3:**
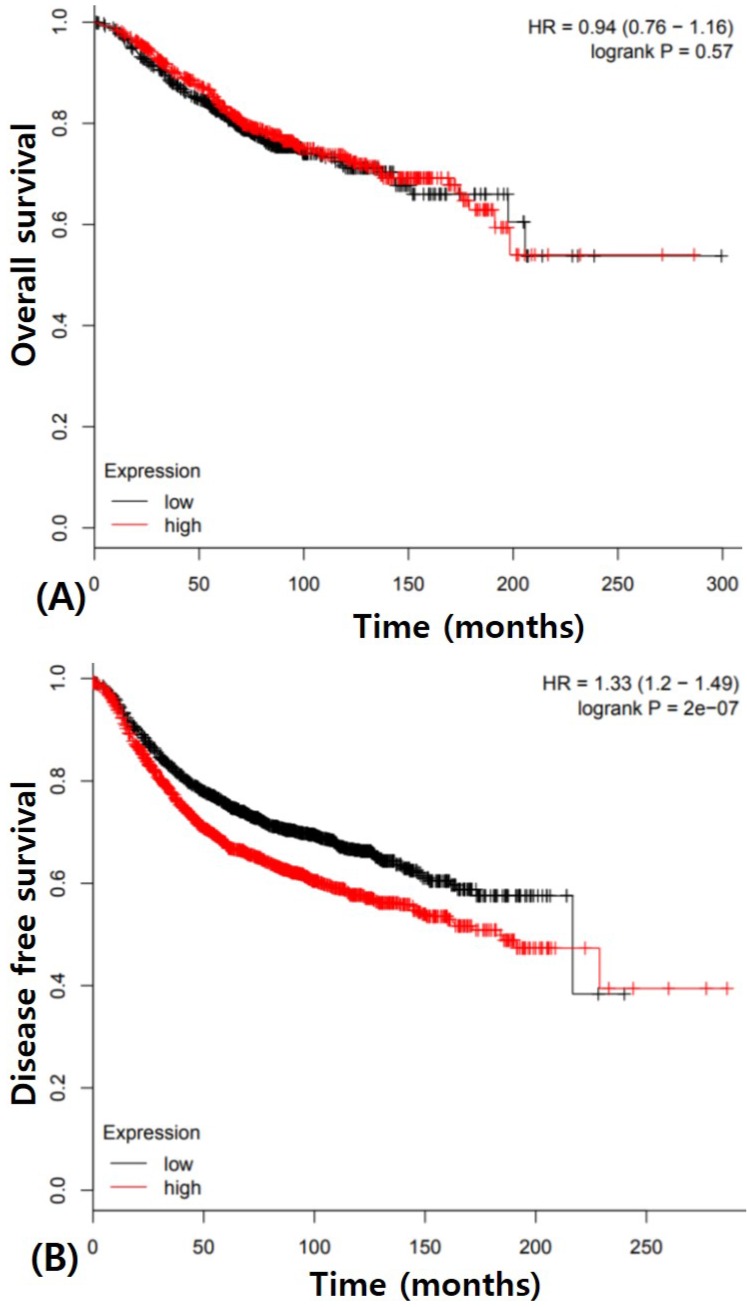
Survival analysis in breast cancers according to PIK3CA expression using The Cancer Genome Atlas (TCGA) data (A) Overall survival (B) Disease free survival.

Prognostic value of *PIK3CA* mutation is extremely controversial in IBC though it may potentially be a better prognostic marker 7,9,10,13,16. We also did not get any results about prognostic value of *PIK3CA* in IBC though patients with *PIK3CA* mutation tend to have better prognosis. *PIK3CA* expression also did not have any prognostic potential for overall survival. These results indicated that the change of single *PIK3CA* gene has little effect on the progression of IBC. The Cancer Genome Atlas (TCGA) data analysis showed no significance of overall survival of *PIK3CA* expression in IBC (Figure 3A). However, *PIK3CA* expression was associated with poorer disease free survival (p < 0.0001, Figure 3B). Therefore, the prognostic value of *PIK3CA* expression in IBC should be investigated using integrated and multidisciplinary approaches.

In present study, we identified the clinical and prognostic characteristics of the expression and mutation of *PIK3CA* in Korean patients with IBC. These findings add to the evidence that *PIK3CA* has a potential role in IBC. A precise understanding of the role of *PIK3CA* in IBC development will usher in a new era for breast cancer prevention and therapy.

## Figures and Tables

**Table 1:** Characteristics of PIK3CA mutation and expression in invasive breast carcinomas

	PIK3CA mutation (n, %)			PIK3CA expression (n, %))		
	(+)	(-)	p	High	Low	p
**Total**	15 (14.3)	90 (85.7)		28 (21.9)	100 (78.1)	
**Age**			0.769			0.524
< 60 years	11 (73.3)	59 (65.6)		20 (71.4)	65 (65.0)	
≥ 60 years	4 (26.7)	31 (34.4)		8 (28.6)	35 (35.0)	
**BMI**			0.828			0.198
≤ 25	6 (54.5)	36 (58.1)		15 (68.2)	33 (52.4)	
> 25	5 (45.5)	26 (41.9)		7 (31.8)	30 (47.6)	
**T stage**			0.586			0.893
T1	5 (33.3)	27 (30.0)		8 (28.6)	31(31.0)	
T2	10 (66.7)	57 (63.3)		18 (64.3)	64 (64.0)	
T3/4	0 (0)	6 (6.7)		2 (7.1)	5 (5.0)	
**N stage**			0.242			0.180
N0	7 (46.7)	57 (63.3)		14 (50.0)	65 (65.0)	
N1	7 (46.7)	23 (25.6)		11 (39.3)	22 (22.0)	
N2	1 (6.7)	10 (11.1)		3 (10.7)	13 (13.0)	
**Histological**			0.089			0.479
Well/ Moderate	11 (73.3)	81 (90.0)		24 (85.7)	91 (91.0)	
poor/ undifferentiated	4 (26.7)	9 (10.0)		4 (14.3)	9 (9.0)	
**Luminal**			0.019			0.011
A	12 (80.0)	37 (41.1)		13 (46.4)	36 (36.0)	
B	1 (6.7)	26 (28.9)		4 (14.3)	35 (35.0)	
HER2+	0 (0)	0 (0)		0 (0)	11 (11.0)	
TN	2 (13.3)	27 (30.0)		11 (39.3)	18 (18.0)	

T stage and N stage were based on the The American Joint Committee on Cancer Staging Manual, 7th edition. HER2, human epidermal growth factor receptor 2; TN, triple negative.
